# Integrin α7 expression is increased in asthmatic patients and its inhibition reduces Kras protein abundance in airway smooth muscle cells

**DOI:** 10.1038/s41598-019-46260-2

**Published:** 2019-07-09

**Authors:** Chun Ming Teoh, Sheryl S. L. Tan, Shenna Y. Langenbach, Amanda H. Wong, Dorothy H. J. Cheong, John K. C. Tam, Chih Sheng New, Thai Tran

**Affiliations:** 1 Department of Physiology, Yong Loo Lin School of Medicine, National University of Singapore, Singapore, Singapore; 2 Department of Pharmacology and Therapeutics, and Lung Health Research Centre, University of Melbourne, Melbourne, Australia; 3 Department of Surgery, Yong Loo Lin School of Medicine, National University of Singapore, Singapore, Singapore

**Keywords:** Asthma, Chronic obstructive pulmonary disease

## Abstract

Airway smooth muscle (ASM) cells exhibit plastic phenotypic behavior marked by reversible modulation and maturation between contractile and proliferative phenotypic states. Integrins are a class of transmembrane proteins that have been implicated as novel therapeutic targets for asthma treatment. We previously showed that integrin α7 is a novel marker of the contractile ASM phenotype suggesting that targeting this protein may offer new avenues to counter the increase in ASM cell mass that underlies airways hyperresponsiveness (AHR) in asthma. We now determine whether inhibition of integrin α7 expression would revert ASM cells back to a proliferative phenotype to cause an increase in ASM cell mass. This would be detrimental to asthmatic patients who already exhibit increased ASM mass in their airways. Using immunohistochemical analysis of the Melbourne Epidemiological Study of Childhood Asthma (MESCA) cohort, we show for the first time that integrin α7 expression in patients with severe asthma is increased, supporting a clinically relevant role for this protein in asthma pathophysiology. Moreover, inhibition of the laminin-integrin α7 signaling axis results in a reduction in smooth muscle-alpha actin abundance and does not revert ASM cells back to a proliferative phenotype. We determined that integrin α7-induced Kras isoform of p21 Ras acts as a point of convergence between contractile and proliferative ASM phenotypic states. Our study provides further support for targeting integrin α7 for the development of novel anti-asthma therapies.

## Introduction

Airway wall remodeling (AWR), a key feature of chronic asthma involves airway smooth muscle (ASM) hypertrophy and hyperplasia and increased deposition of extracellular matrix (ECM) proteins in the airway. ASM cells exist in two extreme phenotypes: the proliferative phenotype and the contractile phenotype^1^. The proliferative phenotype is characterized by the expression of numerous organelles for protein and lipid synthesis, high proliferative rate and low contractile protein expression. In contrast, the contractile phenotype is characterized by low proliferative rate and an increase in the expression of cytoskeletal proteins as well as proteins that are involved in the regulation of ASM contraction signaling, such as smooth muscle (sm)-MHC, sm-α-actin, SM22, desmin and calponin^2,3^. Acquisition of a contractile phenotype is termed maturation, and in cell culture, it can be induced following mitogen withdrawal^3^. There are several signaling molecules that are involved in ASM cell proliferation; namely PI3K, ERK and to some extent, p38 MAPK^4,5^. Cyclin D1 is downstream of PI3K, ERK, and p38 MAPK and is a key regulator of G1-S progression in ASM cells^4^.

Previous studies in our laboratory showed that ASM maturation is achieved through the binding of laminin-211 and integrin α7β1^6^. Laminin, via integrin α7β1 binding, is both necessary and sufficient to promote the accumulation of pro-survival proteins and to reduce the levels of pro-apoptotic proteins in ASM cells. Effects on ASM survival are induced exclusively by laminin-211 and involve signaling pathways that concomitantly regulate ASM cell survival and ASM cell maturation^6^. This suggests that targeting the laminin-211-integrin α7β1 signaling axis may reduce the mass of contractile ASM phenotype cells which is in line with recent studies by others in support of integrins as therapeutic targets in AHR^7^. However, there is a possibility that the inhibition of laminin-integrin binding may induce contractile ASM cells to revert back to their proliferative phenotype. Proliferative ASM phenotype cells may lead to an increase in ASM cell mass, which is detrimental to asthmatic patients who already exhibit increased ASM mass in their airways. Hence, in our current study, we determined whether inhibition of integrin α7β1 expression has an impact on ASM phenotype plasticity by inducing contractile ASM phenotype cells back to a proliferative phenotype and the underlying signaling mechanisms involved in this process.

## Results

### Clinical relevance of integrin α7β1 expression in asthma

**Figure 1:**
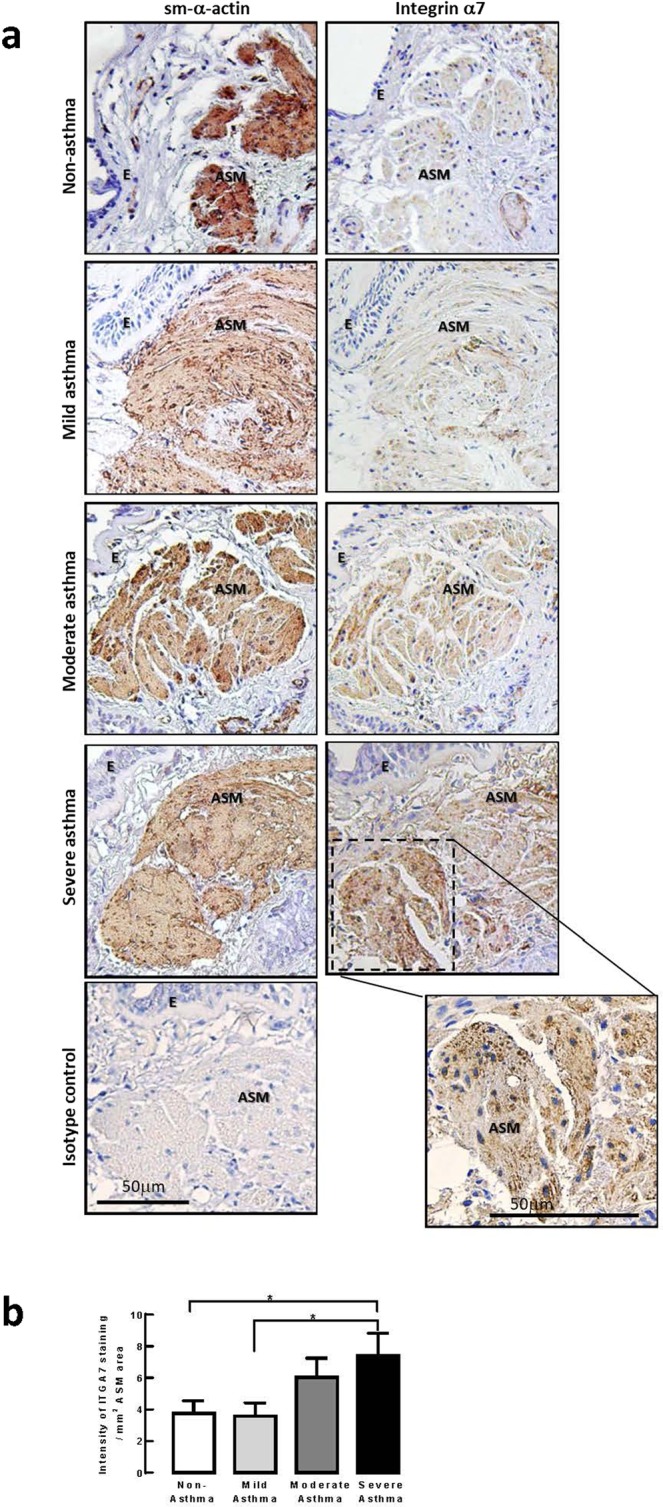
Integrin α7 expression is increased with asthma severity. (**a**) Representative matched immunohistochemical images of human lung biopsies taken from the MESCA (Melbourne Epidemiological Study on Childhood Asthma) cohort stained for smooth muscle (sm)-α-actin and integrin α7. Brown staining represents positive staining for either sm-α-actin or integrin α7, while blue staining represents nuclear staining; ASM = airway smooth muscle, E = epithelium. Bar = 50μm. (**b**) Quantitation of integrin α7 staining intensity in biopsies from subjects with increasing asthma severity. Individual and median values from non-asthma (n = 9), mild asthma (n = 13), moderate asthma (n = 8) and severe asthma (n = 8) subjects are shown. *P < 0.05, Kruskal–Wallis test.

We first determined whether the expression of integrin α7β1 in ASM was clinically relevant by scoring the immunohistochemical staining of integrin α7 in human bronchial biopsies from patients with varying asthma severity (Fig. 1). Histological sections of biopsy samples were taken from an existing collection of biopsy samples that were originally used in a cohort study called MESCA (Melbourne Epidemiological Study of Childhood Asthma)^8–10^. We showed that there was a significant increase in staining intensity for integrin α7 (per mm^2^ ASM area) in subjects with severe asthma as compared with non-asthma subjects (Fig. 1).

### Inhibition of laminin or integrin α7β1 does not revert ASM cells towards increased proliferation

**Figure 2:**
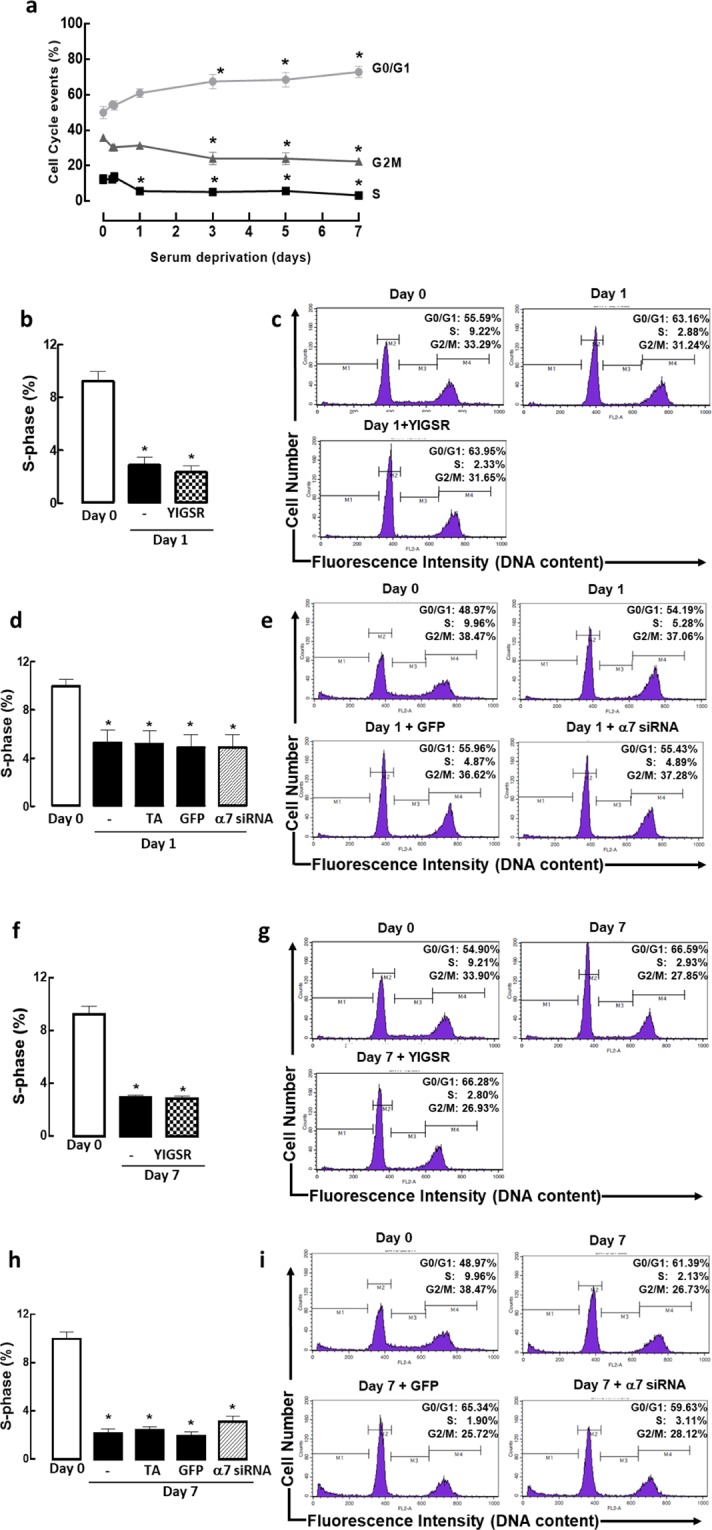
Inhibition of laminin-binding or laminin receptor expression (integrin α7β1) maintains the ASM cells in a low proliferative state. (**a**) Time course and representative histograms showing a typical distribution of cells in G0/G1, S, and G2/M phases with increasing days of serum deprivation. Grouped (**b**) and representative histograms (**c**) showing the effect of the laminin-selective competing peptide (YIGSR, 10 μM) on ASM cell cycle S-phase following day 1 serum deprivation. Grouped (**d**) and representative histograms (**e**) showing the effect of integrin α7 siRNA (1 μM) on ASM cell cycle S-phase following day 1 serum deprivation. Grouped (**f**) and representative histograms (**g**) showing the effect of the laminin-selective competing peptide (YIGSR, 10 μM) on ASM cell cycle S-phase following day 7 serum deprivation. Grouped (**h**) and representative histograms (**i**) showing the effect of integrin α7 siRNA (1 μM) on ASM cell cycle S-phase following day 7 serum deprivation. Transfection agent (TA) served as vehicle control, green fluorescence protein (GFP) siRNA served as negative control. Results are representative of 3 independent experiments. *P < 0.05 compared with Day 0. Portions of this study have been deposited in scholarbank.nus.edu.sg^35^.

Proliferative ASM phenotype cells are characterized by high proliferative rate whereas contractile ASM phenotype cells are characterized by low proliferative rate. A time course experiment was carried out to determine the period in which ASM cells in S-phase of the cell cycle was reduced during the 7-day serum deprivation. The percentage of cells in the S-phase was used as a proliferative index in this study. ASM cells in S-phase reduced significantly after day 1 serum deprivation and this reduction was sustained for up to 7 days of serum deprivation (Fig. 2a and Supplementary Fig. 1). With this, day 1 serum deprivation was selected for subsequent experiments with the treatment of a laminin-selective competing peptide, YIGSR or integrin α7β1 siRNA. There was no significant change in the population of ASM cells in S-phase with the treatment of laminin-selective competing peptide (Fig. 2b,c) or knockdown of integrin α7β1 (Fig. 2d,e). Consistent with this, there was no significant change in the population of ASM cells in S-phase with both YIGSR and integrin α7β1 siRNA treatment across day 3 (Supplementary Fig. 2) and day 7 (Fig. 2f–i).

**Figure 3:**
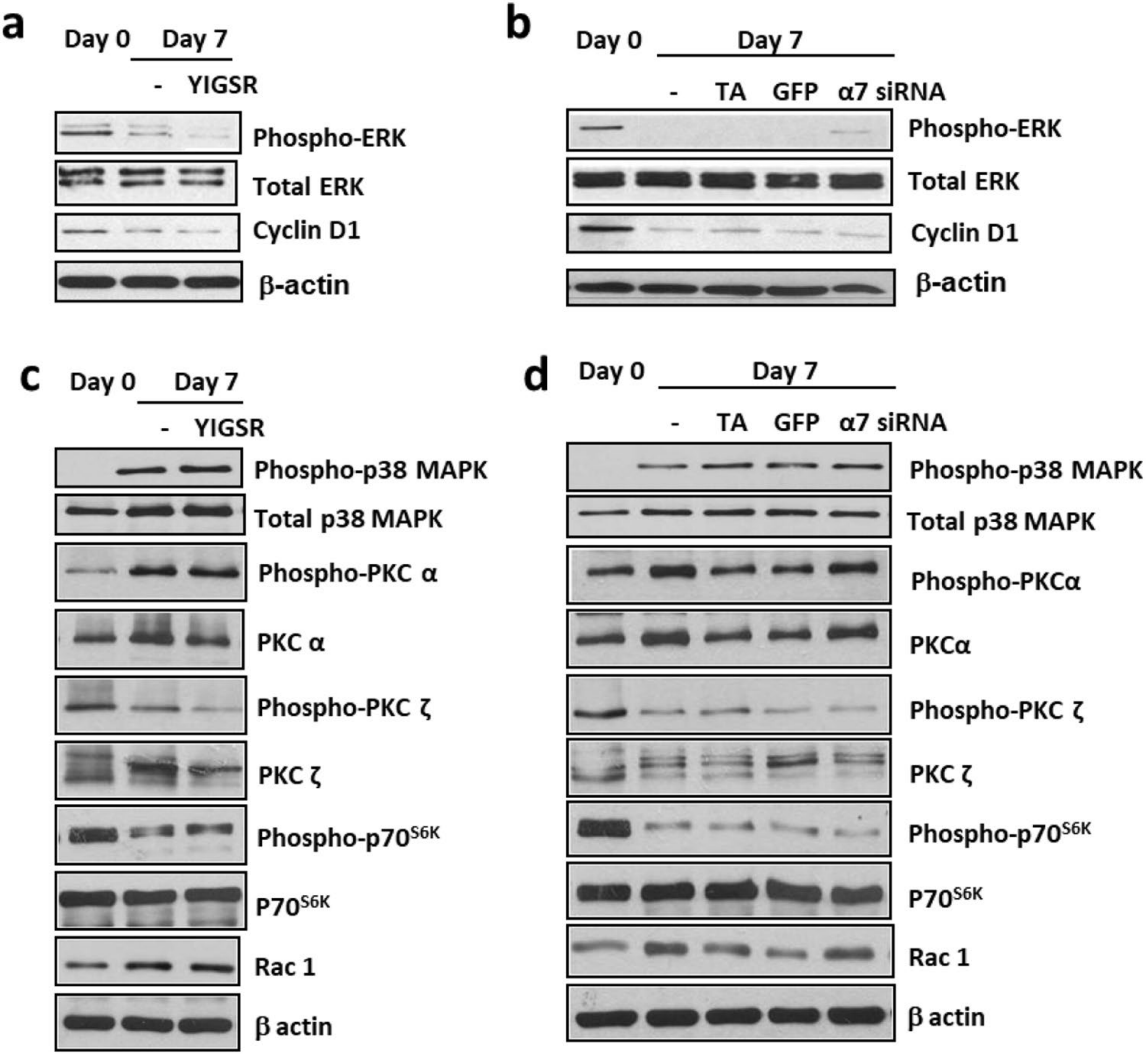
Laminin and integrin α7β1 are not required for ERK, cyclin D1, p38 MAPK, PKC, p70s6K, or Rac1 protein abundance. Effect of laminin-selective competing peptide (YIGSR, 10 μM, (**a**) or integrin α7 siRNA (1 μM, (**b**) treatment on ERK and cyclin D1 protein abundance. Effect of laminin-selective competing peptide (YIGSR, 10 μM, (**c**) or integrin α7 siRNA (1 μM, (**d**) on p38 MAPK, PKC, p70s6K, and Rac1 protein abundance.  Transfection agent (TA) served as vehicle control, green fluorescence protein (GFP) siRNA served as negative control. Data are expressed as fold increment over basal (Day 0) relative to β-actin protein abundance. Results are representative of 3 independent experiments. *P < 0.05 compared with Day 0; ^†^*P* < 0.05 compared with Day 7 response without YIGSR or integrin α7 siRNA.

To confirm the above cell cycle analysis results, we investigated the protein abundance of ERK and cyclin D1, which are markers of cell proliferation^11,12^. Indeed, contractile ASM phenotype cells (Day 7) exhibited lower levels of phospho-ERK and cyclin D1 proteins compared to control (Day 0) (Fig. 3a). The laminin-selective competing peptide, YIGSR, did not modulate the expression levels of these two proteins. Moreover, silencing of integrin α7β1 only partially reversed phospho-ERK protein abundance (Fig. 3b) and no changes were seen in cyclin D1 protein expression (Fig. 3b). This further suggests that laminin and integrin α7β1 are not required for ERK and cyclin D1 protein expression and that laminin inhibition maintains ASM cells in a low proliferative state.

p38 MAPK has been shown to negatively regulate cyclin D1 expression in ASM cells^13,14^. When we induced ASM cells to the contractile phenotype (Day 7), phospho-p38 MAPK protein abundance was significantly increased compared to cells exhibiting the proliferative phenotype (Day 0) (Fig. 3c,d). In contrast, treatment of ASM cells with YIGSR or integrin α7β1 siRNA had no effect on phospho-p38 MAPK protein abundance (Fig. 3c,d). This suggests that p38 MAPK is not affected by laminin or integrin α7β1 inhibition.

Another important pathway regulating ASM cell proliferation is the PI3K pathway^4^. Our previous studies showed that treatment of ASM cells with YIGSR or integrin α7β1 siRNA reduced the activation of Akt; where Akt is the main substrate of PI3K required for ASM cell maturation and survival^6^. Thus, we were interested to investigate the effect of laminin inhibition on proteins involved in ASM proliferation signaling that was downstream of PI3K. These proteins include phospho-p70^S6K^, Rac1, phospho-PKCα and phospho-PKCς. In the presence of laminin-selective competing peptide, YIGSR, or integrin α7β1 siRNA, the levels of phospho-p70^S6K^, Rac1, phospho-PKCς and phospho-PKCα proteins were unchanged (Fig. 3c,d). This suggests that laminin and integrin α7β1 are not required for phospho-p70^S6K^, Rac1, phospho-PKCα and phospho-PKCς protein abundance. Phospho-p70^S6K^, Rac1, phospho-PKCα and phospho-PKCς are proteins downstream of the PI3K pathway that are involved in ASM cell proliferation signaling. The lack of effect on these proteins following YIGSR or integrin α7β1 siRNA treatment suggests that the inhibition of laminin-integrin binding has little impact on reversing ASM cells to a proliferative phenotype.

### Integrin α7β1 is not required for bFGF- or FBS-induced ASM proliferation

**Figure 4:**
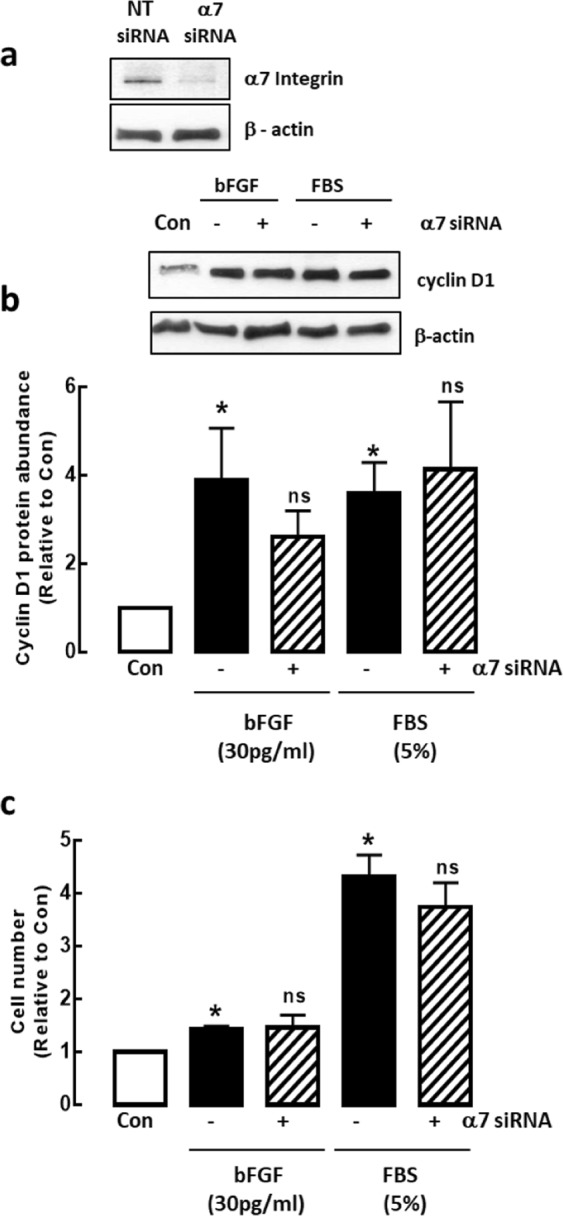
(**a**) Validation of integrin α7 siRNA on integrin α7 protein abundance. NT = non-targeting siRNA. Effect of integrin α7 siRNA on bFGF or FBS-induced increases in (**b**) cyclin D1 protein abundance following 20 hr mitogen stimulation, and (**c**) cell number following 48 hr mitogen stimulation. Data are expressed as fold increment over control (Day 0). Results are representative of 3–5 independent experiments. *P < 0.05 compared with control; ns = not significant compared with respective mitogen.

The above experiments demonstrated that inhibition of integrin α7β1 does not revert ASM cells (Day 7 cells) back to a proliferative phenotype as measured by lack of changes in proliferative makers. To further support that integrin α7β1 does not act on ASM proliferative phenotype, we also determined the effects of two mitogens in the presence and absence of integrin α7 siRNA treatment (Fig. 4). As expected, stimulation with either bFGF or FBS increased cyclin D1 protein abundance (20 hrs) and cell number (48 hrs) relative to unstimulated cells (Day 0). In the presence of integrin α7 siRNA, cyclin D1 protein abundance and cell number was not reduced (Fig. 4).

### Laminin or integrin α7β1-induced Kras protein abundance acts as a point of convergence between contractile and proliferative ASM phenotype states

**Figure 5:**
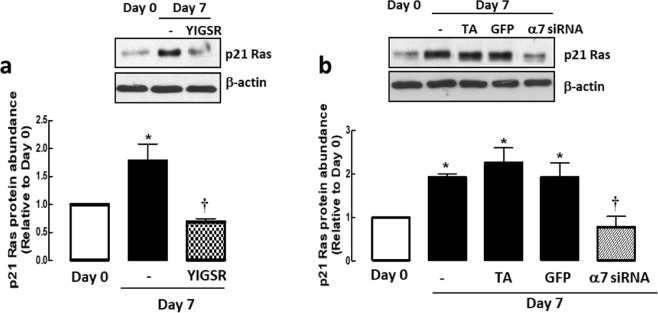
Laminin-binding (**a**) and integrin α7β1 (**b**) expression is required for p21 Ras protein abundance. Transfection agent (TA) served as vehicle control, green fluorescence protein (GFP) siRNA served as negative control. Data are expressed as fold increment over basal (Day 0) relative to β-actin protein abundance. Results are representative of 3 independent experiments. *P < 0.05 compared with Day 0; ^†^*P* < 0.05 compared with Day 7 response without YIGSR or integrin α7 siRNA.

Having shown that laminin inhibition did not affect ERK, proteins downstream of PI3K and p38 MAPK protein abundance, we looked upstream to identify possible proteins that might regulate ASM phenotypic maturation whilst maintaining ASM cells at a low proliferative index. p21 Ras is a protein that is upstream of ERK, PI3K and p38 MAPK^4,5,13^. By western blot, we showed that both laminin inhibition and silencing of integrin α7β1 reduced p21 Ras protein expression significantly (Fig. 5), suggesting that p21 Ras protein is laminin- and integrin α7β1-dependent.

**Figure 6:**
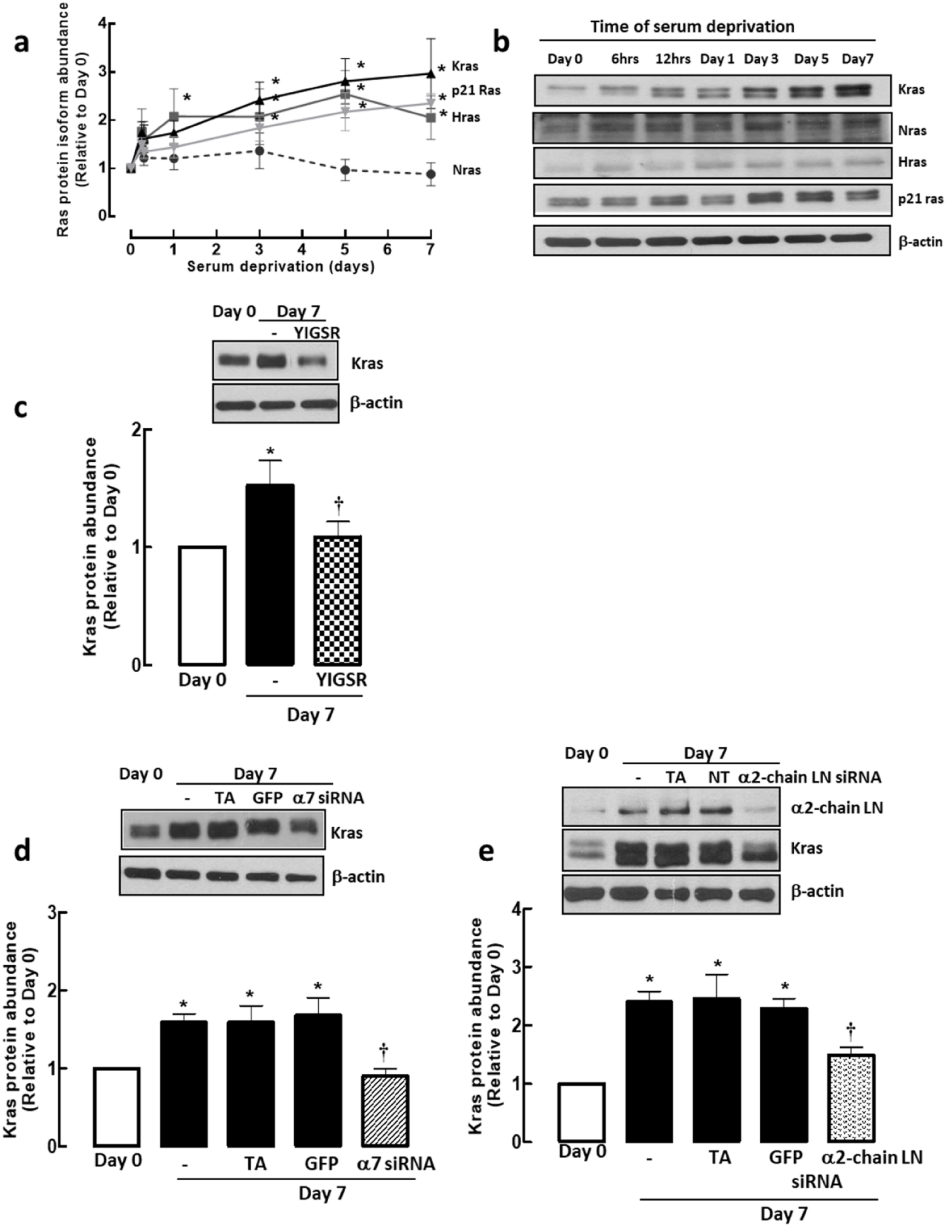
Grouped (**a**) and representative western blots (**b**) of time course experiments showing the typical distribution of p21 Ras protein isoforms with increasing days of serum deprivation. (**c**) Laminin-binding and (**d**) integrin α7β1 expression are required for Kras protein abundance. (**e**) Effect of α2-chain laminin siRNA (50 nM) on Kras protein abundance. Transfection agent (TA) served as vehicle control, green fluorescence protein (GFP) and non-targeting (NT) siRNAs served as negative control, LN, Laminin. Results are representative of 3 independent experiments. Data are expressed as fold increment over basal (Day 0) relative to β-actin protein abundance. *P < 0.05 compared with Day 0; ^†^*P* < 0.05 compared with Day 7 response without YIGSR, integrin α7 siRNA or α2-chain laminin siRNA.

We further looked to determine the mechanism by which laminin regulates p21 Ras protein abundance with time and showed that of the 3 isoforms of p21 Ras, only Hras and Kras protein were upregulated with increasing days of serum deprivation; the protein levels of Nras remained the same throughout the same period (Fig. 6a,b). p21 Ras and Kras peaked at day 3 serum deprivation whereas Hras started to peak after day 1 serum deprivation. The same transition time between p21 Ras and Kras suggests that the reduction in total p21 Ras protein expression with laminin-competing peptide and integrin α7β1 siRNA treatments previously may be due to Kras instead of Hras. To confirm this, ASM cells were treated with YIGSR or integrin α7β1 siRNA for 7-day serum deprivation. YIGSR or integrin α7β1 siRNA treated cells showed lower expression of Kras compared with Day 7 cells (Fig. 6c,d). In contrast, the expression of Nras and Hras remained the same even in the presence of YIGSR or integrin α7β1 siRNA. From our previous studies, we showed that all the chains that constitute laminin-211 were elevated^15^. To further confirm that laminin is responsible for the observed effects, we performed the same experiments with α2-chain laminin siRNA. Using this approach, we observed that by reducing α2-chain laminin protein, we also suppressed Kras accumulation (Fig. 6e).

**Figure 7:**
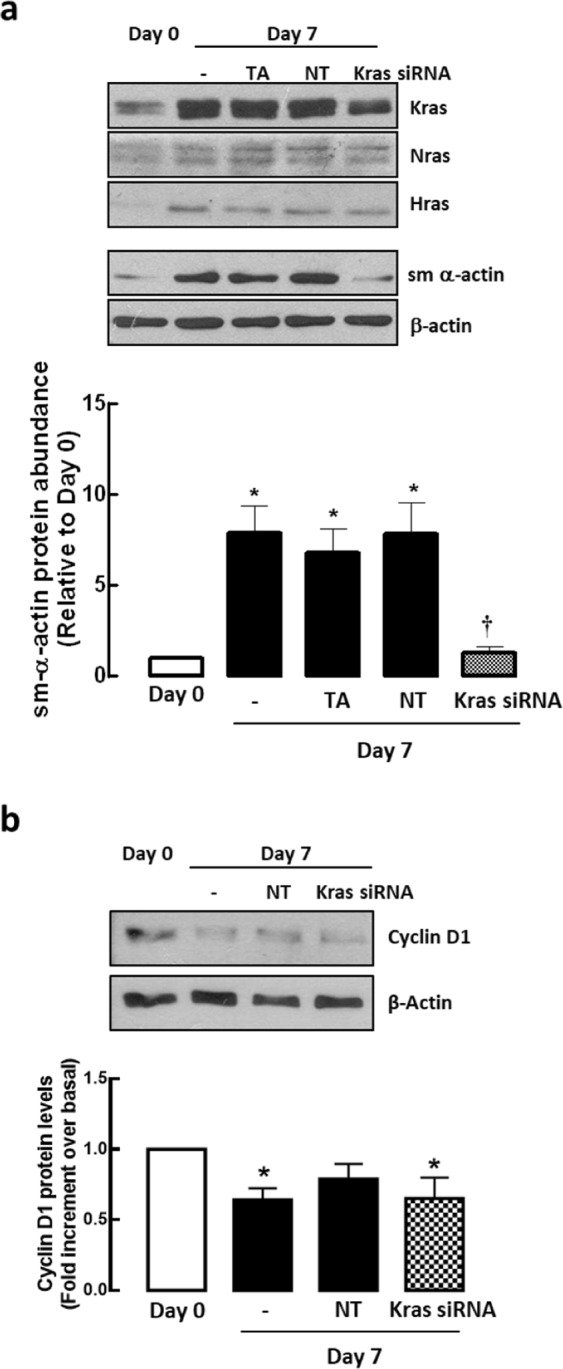
Silencing of Kras protein markedly reduces sm-α-actin expression with no change in cyclin D1 protein abundance. Effect of Kras siRNA (50 nM) on (**a**) p21 Ras isoforms, sm-α-actin, and (**b**) cyclin D1 protein abundance. Transfection agent (TA) served as vehicle control, non-targeting (NT) siRNA served as negative control. Results are representative of 4–6 independent experiments. Data are expressed as fold increment over basal (Day 0) relative to β-actin protein abundance. *P < 0.05 compared with Day 0; ^†^*P* < 0.05 compared with Day 7 response without Kras siRNA.

As a proof-of-concept study, we ascertained the effect of inhibition of Kras expression on sm-α-actin abundance. Silencing of Kras protein markedly reduced sm-α-actin protein abundance in contractile ASM phenotype cells (Day 7) (Fig. 7a) and had very little impact on cyclin D1 protein levels in these cells (Fig. 7b).

## Discussion

We previously showed that laminin-induced ASM cell maturation (induction to a contractile phenotype) is linked to increased ASM survival signalling^6^. An increase in contractile ASM cells over time may contribute to AWR and AHR. We now extend these studies to show that integrin α7 protein expression is significantly increased in patients with severe asthma and that integrin α7β1 blockade not only reduces contractile ASM phenotype (ie. reduction in sm-α-actin) but also prevents their conversion back to a proliferative phenotype via a Kras-dependent mechanism.

Various integrins have been implicated to modulate aspects of asthma pathophysiology - airway inflammation^7^, AWR^16^, and AHR^7^. Hence, it has been proposed that integrins are therapeutic targets in asthma. However, the expression levels of integrins in relation to asthma severity are not fully characterized. In the present study, we characterize for the first time, the expression levels of integrin α7 in lung biopsies of asthmatic patients. We show that with increasing severity of asthma, there is a concomitant increase in integrin α7 expression. This data strongly supports the clinical importance of integrin α7 in asthma and the need to further examine its contribution to asthma pathogenesis. Integrin α7 complexes with integrin β1. Although integrin β1 subunit induces downstream signaling events to regulate actin cytoskeletal dynamics and cell cycle progression^17^, we did not look at integrin β1 expression levels in these samples as it is the binding of the integrin α subunit with the ECM that directs the downstream events evoked by integrin β1. Hence, it is changes in the expression levels of the integrin α subunit that is critical in directing cell-specific responses^17,18^. Consistent with this, our previous studies in cell culture showed no difference in integrin β1 expression levels between ASM cells of the proliferative versus contractile phenotype^19^.

Phenotypic plasticity of ASM is thought to promote fibroproliferative disorders such as asthma. The ability of ASM cells to switch between the proliferative and contractile phenotypes may be governed by a variety of growth factors and ECM proteins. Extensive studies have been done to characterize the effects of ECM proteins on ASM cell phenotype and function^20–24^. However, limited studies have been done on the functional consequences of ECM protein inhibition on ASM cells. This is the first study to demonstrate that laminin inhibition does not revert ASM cells back to its proliferative state and we confirm this by use of two tools: laminin competing peptide, YIGSR and integrin α7β1 siRNA. ASM cells remained quiescent with a low percentage of cells in the S-phase of the cell cycle with YIGSR and integrin α7β1 siRNA treatments respectively. Protein markers of ASM proliferation were also not affected. Our observations are significant because they reveal that blocking of endogenous laminin or its corresponding binding-receptor, integrin α7β1, does not enhance ASM proliferation. Our findings are consistent with a study done by Vukicevic and colleagues^25^. They showed that differentiation of rat primary calvarial bone cells, characterized by reduced cell growth, was influenced by laminin^25^. This differentiation process was blocked by YIGSR-NH_2_ in a dose-dependent manner but there was no influence on cell proliferation. However, when the cells were treated with other laminin synthetic peptides such as RGD or IKVAV, the effects were different. RGD had no influence on blocking differentiation whereas cells treated with IKVAV stimulated proliferation of the cells^25^. This suggests that laminin has multiple active sites and thus various receptor-regulated-cell-specific intracellular events. Blocking the binding of specific active sites of laminin may be critical for clinical use^26^. Although we found that there was a partial increase in ERK activation after integrin α7β1 knockdown, it was not sufficient to increase cyclin D1 protein expression, a protein that is downstream of ERK and important for cell cycle progression from G1 to S phase^11,12^. This is in contrast to the work done by Flintoff-Dye and colleagues. They showed that the loss of integrin α7β1 resulted in increased activation of ERK, leading to skeletal muscle cell hyperplasia^27,28^. The apparent discrepancy between our findings with that of Flintoff-Dye and colleagues could possibly be explained by differences in cell type (skeletal versus ASM cells) and thus the signaling mechanism(s) regulating muscle proliferation may be different. We performed our experiments using non-asthmatic cells as a simple model to determine the effect of inhibition of integrin α7 on the proliferative capacity of ASM cells in culture. Our current study would be further strengthened if the same experiments were carried out in asthmatic ASM cells. A better model would be to examine this in a whole animal system instead of individual cells in future studies. In support, using a guinea pig model of chronic asthma, Dekkers and colleagues showed that treatment with YIGSR fully reversed the expression levels of the proliferative marker, PCNA, in ovalbumin-challenged mice^16^.

In our study, we were able to show that all three protein isoforms of p21 Ras: Kras, Nras and Hras were detected in ASM cells in culture. This is in contrast to a study done by Ammit and colleagues^29^. They showed that ASM cells expressed Kras and Nras but not Hras. This may be attributed to the differences in experimental protocols. Ammit and colleagues measured p21 Ras isoforms after 1 hour of EGF, thrombin or bradykinin stimulation. In our study, we measured p21 Ras protein isoforms after 7-day serum deprivation. This suggests that 1 hour may not be sufficient to induce Hras protein expression in ASM cells in their experimental model as compared with our 7-day serum deprivation protocol. In any case, it is clear from the literature that mutation of the Kras gene is implicated in the development of many human cancers such as colorectal and lung cancers. This has triggered intensive efforts in developing effective therapies to treat Kras-driven cancers. However, inhibiting the protein directly has met with limited success. Results from our current study support the notion for targeting laminin-integrin α7 binding signaling as an alternative strategy to reduce Kras accumulation. Indeed, the interaction between laminin and cancer cells is a key event in tumor invasion and metastasis^30^. Importantly, we determined that p21 Ras appears to not only act as the point of convergence for diverse receptor-operated mechanisms of ASM proliferation^4^ but that in particular, Kras isoform of p21 Ras, also acts as a point of convergence between maturation and modulation of ASM cells.

We have shown that the inhibition of the laminin-211-integrin α7β1 signaling axis does not revert contractile ASM phenotype cells back to a proliferative phenotype. This is clinically important as therapies targeting this signaling pathway would reduce contractile ASM cells, but not increase ASM proliferation. Moreover, the observation that integrin α7β1 expression on ASM is significantly higher in severe asthmatics compared to non-asthmatic patients lend further support for targeting the integrin α7β1 signaling pathway for the development of novel anti-asthma therapies.

## Methods

### Subjects

Subjects, either non-asthmatic or asthmatic volunteers with varying asthma severities (as classified by Global Initiative for Asthma guidelines), were recruited from the Melbourne Epidemiological Study of Childhood Asthma (MESCA) cohort with informed consent and with approval from the Human Experimental Ethics Committees of the Royal Children’s and Royal Melbourne Hospitals. All subjects were aged 42 years at the time of biopsy, as previously described^8–10^. Further demographic data are outlined in Table 1. All methods were performed in accordance with the Institutional Biosafety Committee at the National University of Singapore (NUS).

**Table 1:** Demographic data for subjects from the MESCA cohort from whom biopsies were obtained for immunohistochemistry.

	Non-asthma	Mild asthma	Moderate asthma	Severe asthma
Subjects (n)	9	13	8	8
Gender (M/F)	4/5	7/6	6/2	2/8
Atopic (%)	40%	85%	100%	50%
FEV_1_ (mean ± SEM)	109 ± 5	101 ± 3	62 ± 3*	68 ± 5*
Current smokers (%)	70%	54%	38%	0%
Current treatment (%)				
β_2_- agonists	0%	62%	100%	100%
Inhaled steroids	0%	31%	100%	100%
Oral steroids	0%	15%	25%	100%

### Immunohistochemistry

For the MESCA biopsy cohort samples^8–10^, three-micron biopsy sections were stained for integrin α7 (ITGA7, 1:350 dilution) (Sigma, Saint Louis, MO, USA) and sm-α-actin (1:500 dilution) (Dako, Denmark). The intensity of ITGA7 in the smooth muscle was measured, using the cellSens dimension software (Olympus, Germany) at a magnification of 20x and expressed per mm^2^ of sm-α-actin positive biopsy area^31^. Staining, image capture, and measurements were taken by a single observer in a blinded manner.

### Cell culture

Human ASM cell lines were generated using MMLV retroviral transfection to facilitate stable integration of the human telomerase reverse transcriptase (hTERT) gene as previously described^32^ (gift from Professor Andrew Halayko, University of Manitoba, Canada). hTERT-expressing human ASM cell lines retain the ability to express contractile ASM phenotype markers which include sm-MHC, calponin, desmin and sm-α-actin^15,33^. hTERT-expressing human ASM cell lines between passage 29 and 39 were used. To induce a contractile phenotype, cells were serum deprived in Dulbecco’s Modified Eagle’s Medium (DMEM) with 1% ITS (insulin 5 μg/ml; transferrin 5 μg/ml; selenium 5 μg/ml) for up to 7 days^31^.

### Laminin-competing peptide (YIGSR) and siRNA preparation

The laminin-selective competing pentapeptide, YIGSR, corresponds to the 929–933 sequence of the β chain of laminin^34^ and is found to compete with laminin for binding to the laminin receptor. The YIGSR peptide (Sigma, Saint Louis, MO) was reconstituted in distilled water to a stock concentration of 10 mM and then diluted to 1 μM final concentration in serum-free DMEM for use in experiments as previously described^15^. Primers that amplified integrin α7 cDNA were used to prepare siRNA (Gene Therapy System, San Diego, CA) as previously described^31^. Kras and α2-chain laminin-specific ON-TARGETplus SMARTpool siRNAs and ON-TARGETplus non-targeting siRNA (NT siRNA) were purchased from Dharmacon (Thermo Fisher, Rockford, IL, USA). Each ON-TARGETplus SMARTpool siRNA contains a mixture of 4 SMARTselection-designed siRNAs targeting the Kras and LAMA-2 genes respectively. siRNA against integrin α7 (1 μM)^19^, Kras (50 nM) and α2-chain laminin (50 nM)^6^, and YIGSR (10 μM)^15^ were added at the time of serum deprivation and added again when serum deprivation exceeded three days.

### Immunoblotting

ASM cells were lysed and the protein*s* (10–12 µg) were resolved on a 10–12% SDS-PAGE, then transferred onto nitrocellulose membranes (Bio-Rad, USA) as previously described^31^. Antibodies used were sm-α-actin (1:3000), β-actin (1:15000), α2-chain laminin (1:500), Hras (1:200), Kras (1:300), Nras (1:500) (all from Sigma, USA); phospho-ERK, total ERK, phospho-p38 MAPK, total p38 MAPK, p21 Ras, phospho-PKCα, total PKCα, phospho-PKCς, total PKCς, phospho-p70^S6K^, total p70^S6K^, cyclin D1 (all 1:1000) (all from Cell Signaling, USA); and integrin α7 (1:500) (Abcam, Cambridge, UK). Proteins were visualized on Kodak film after incubation with enhanced chemiluminescence reagents, then exposure levels were quantified by TotalLab (UK) densitometry software. Results were expressed as fold increment over Day 0 relative to β-actin.

### Cell cycle analysis

ASM cells were fixed with 70% ethanol and resuspended in 5 µg/ml propidium iodide (PI) solution containing 0.1% Triton X-100, and 50 µg/ml RNase. Cell cycle analysis was assessed using a BD FACScalibur flow cytometer (BD Biosciences, California, USA). Fluorescence histograms were collected and analyzed for at least 10,000 cells, using CellQuest Pro software (BD Biosciences, California, USA).

### Statistical analysis

Statistical analysis was performed with GraphPad Prism 6 (GraphPad, San Diego, CA, USA) using either one-way ANOVA with repeated measures, followed by Bonferroni’s *post hoc t*-test for the cell line studies and Kruskal–Wallis test for the MESCA samples. A probability value of *P* < 0.05 was considered significant.

## Supplementary information

Supplementary 1–2

## Data Availability

No datasets were generated or analyzed during the current study.
