# Supplementary material for: Integrin α7 expression is increased in asthmatic patients and its inhibition reduces Kras protein abundance in airway smooth muscle cells
Source: Sci Rep. 2019 Jul 9;9:9892. doi: 10.1038/s41598-019-46260-2 (PMC6616330; doi:10.1038/s41598-019-46260-2)
Supplement: Supplementary file 1 — Supplementary 1–2 [file 41598_2019_46260_MOESM1_ESM.pdf]

Integrin α7 expression is increased in asthmatic patients and its inhibition reduces Kras protein abundance in airway smooth muscle cells

Chun Ming TEOH[1§], Sheryl S.L. TAN[1§], Shenna Y. LANGENBACH[2], Amanda WONG[1], Dorothy CHEONG[1], John Kit Chung TAM[3], ChihSheng NEW[1,] and Thai TRAN[1,*]

[§] Both authors contributed equally

[1]Department of Physiology, Yong Loo Lin School of Medicine, National University of Singapore, Singapore

[2]Department of Pharmacology and Therapeutics, and Lung Health Research Centre, University of Melbourne, Australia

[3]Department of Surgery, Yong Loo Lin School of Medicine, National University of Singapore, Singapore

* Corresponding author:

Thai Tran, PhD

Department of Physiology,

MD9, 2 Medical Drive,

National University of Singapore, Singapore 117593

Tel: +65 6516-3663;

Email: phstt@nus.edu.sg

SUPPLEMENTARY

Figure 1: Representative histograms showing a typical distribution of cells in G0/G1, S, and G2/M phases with increasing days of serum deprivation. Results are representative of 3 independent experiments.

Figure 2: Representative histograms showing the effect of **(a)** laminin-selective competing peptide (YIGSR, 10μM) or **(b)** integrin α7 siRNA (1μM) on ASM cell cycle S-phase following day 3 serum deprivation. Green fluorescence protein (GFP) siRNA served as negative control. Results are representative of 3 independent experiments.

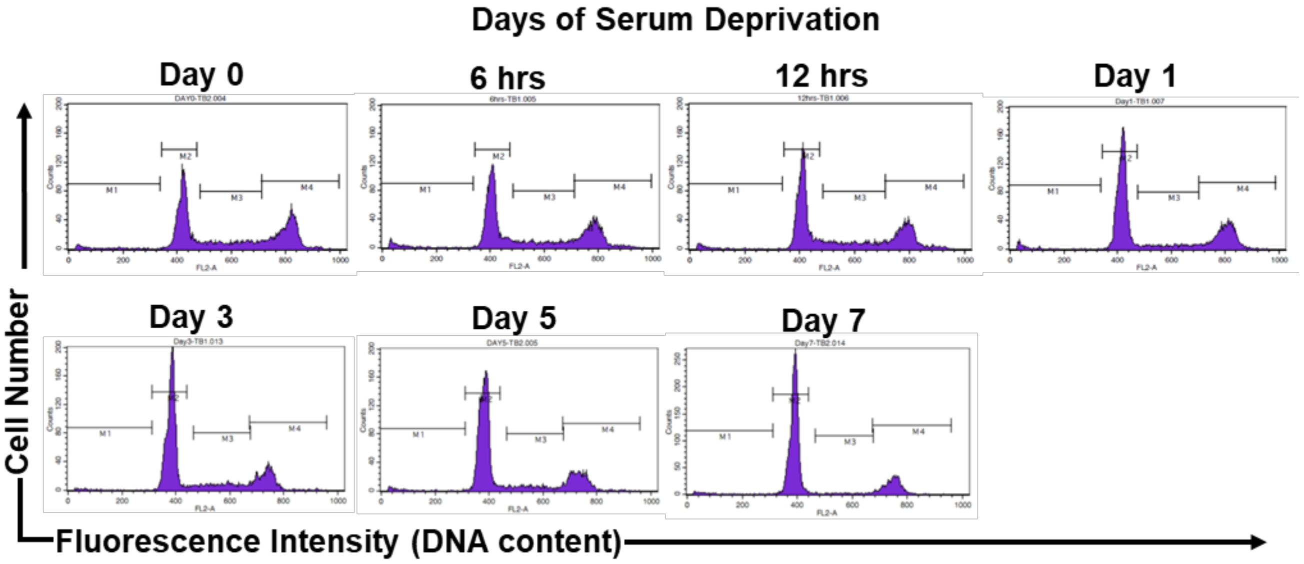
Days of Serum Deprivation
Day 0
6 hrs
12 hrs
Day 1
Day 3
Day 5
Day 7
Cell Number
Fluorescence Intensity (DNA content)

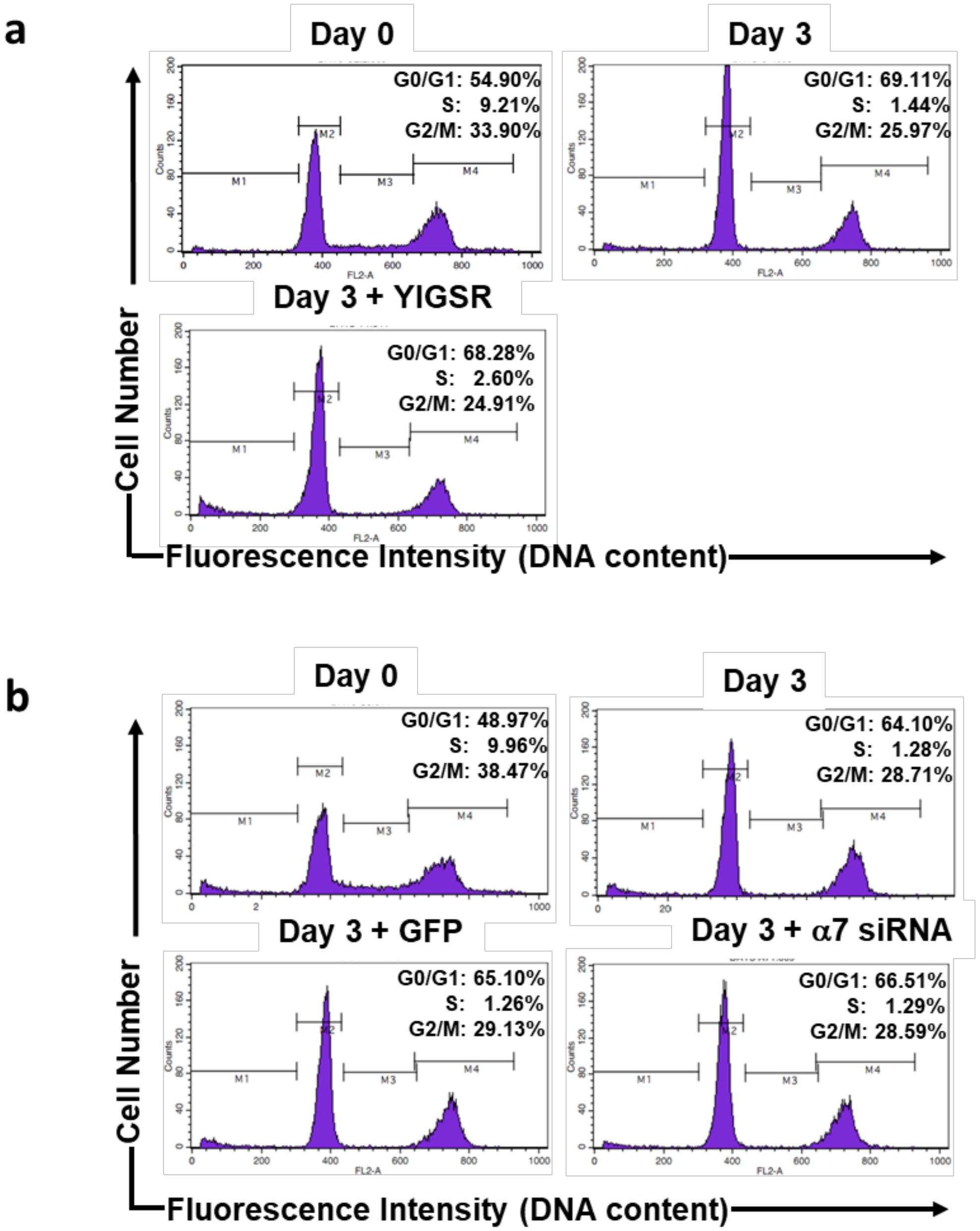

a
Day 0
G0/G1: 54.90%
S: 9.21%
G2/M: 33.90%
Day 3
G0/G1: 69.11%
S: 1.44%
G2/M: 25.97%
Day 3 + YIGSR
G0/G1: 68.28%
S: 2.60%
G2/M: 24.91%
Cell Number
Fluorescence Intensity (DNA content)
b
Day 0
G0/G1: 48.97%
S: 9.96%
G2/M: 38.47%
Day 3
G0/G1: 64.10%
S: 1.28%
G2/M: 28.71%
Day 3 + GFP
G0/G1: 65.10%
S: 1.26%
G2/M: 29.13%
Day 3 + α7 siRNA
G0/G1: 66.51%
S: 1.29%
G2/M: 28.59%
Cell Number
Fluorescence Intensity (DNA content)
